# Phylosymbiosis Impacts Adaptive Traits in *Nasonia* Wasps

**DOI:** 10.1128/mBio.00887-19

**Published:** 2019-07-16

**Authors:** Edward J. van Opstal, Seth R. Bordenstein

**Affiliations:** a Department of Biological Sciences, Vanderbilt University, Nashville, Tennessee, USA; b Vanderbilt Microbiome Initiative, Vanderbilt University, Nashville, Tennessee, USA; c Department of Pathology, Microbiology, and Immunology, Vanderbilt University, Nashville, Tennessee, USA; d Vanderbilt Institute for Infection, Immunology, and Inflammation, Vanderbilt University, Nashville, Tennessee, USA; Christian-Albrechts University-Kiel; New York University

**Keywords:** *Nasonia*, evolution, microbiome, phylosymbiosis

## Abstract

Phylosymbiosis is defined as microbial community relationships that recapitulate the phylogeny of hosts. As evidence for phylosymbiosis rapidly accumulates in different vertebrate and invertebrate holobionts, a central question is what evolutionary forces cause this pattern. We use intra- and interspecific gut microbiota transplants to test for evidence of selective pressures that contribute to phylosymbiosis. We leverage three closely related species of the parasitoid wasp model *Nasonia* that recently diverged between 0.4 and 1 million years ago: N. vitripennis, N. giraulti, and N. longicornis. Upon exposure of germfree larvae to heat-inactivated microbiota from intra- or interspecific larvae, we measure larval growth, pupation rate, and adult reproductive capacity. We report three key findings: (i) larval growth significantly slows when hosts receive an interspecific versus intraspecific gut microbiota, (ii) marked decreases in pupation and resulting adult survival occur from interspecific gut microbiota exposure, and (iii) adult reproductive capacities including male fertility and longevity are unaffected by early life exposure to an interspecific microbiota. Overall, these findings reveal developmental and survival costs to *Nasonia* upon larval exposures to interspecific microbiota and provide evidence that selective pressures on phenotypes produced by host-microbiota interactions may underpin phylosymbiosis.

## Abstract (precis)

Phylosymbiosis is an ecoevolutionary hypothesis and emerging pattern in animal-microbiota studies whereby the host phylogenetic relationships parallel the community relationships of the host-associated microbiota. A central prediction of phylosymbiosis is that closely related hosts exhibit a lower microbiota beta diversity than distantly related hosts. While phylosymbiosis has emerged as a widespread trend in a field often challenged to find trends across systems, two critical and understudied questions are whether or not phylosymbiosis is consequential to host biology and if adaptive evolutionary forces underpin the pattern. Here, using germfree rearing in the phylosymbiosis model *Nasonia*, we demonstrate that early life exposure to heat-inactivated microbiota from more distantly related species poses more severe developmental and survival costs than microbiota from closely related or the same species. This study advances a functional understanding of the consequences and potential selective pressures underpinning phylosymbiosis.

## INTRODUCTION

**FIG 1:**
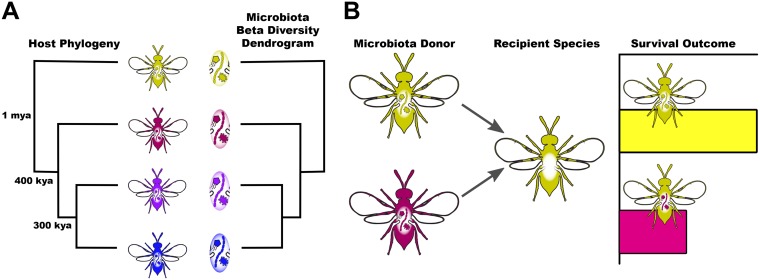
Predictions of phylosymbiosis. (A) The host-associated microbiota is distinguishable between related species, and host phylogenetic relationships parallel the beta diversity relationships depicted by the microbiota dendrogram in this case. Estimated ages of divergence are depicted at phylogenetic nodes. (B) If phylosymbiosis is consequential to host biology (e.g., survival), then introductions of interspecific microbiota will be more costly than introduction of intraspecific microbiota in host recipients.

Phylosymbiosis occurs when host phylogenetic relationships parallel the community relationships of the host-associated microbiota (1, 2). A central prediction of phylosymbiosis is that phylogenetically similar host species will exhibit lower microbiota beta diversity than distantly related hosts, in contrast to the null hypothesis that microbiota beta diversity relationships do not parallel host phylogenetic relationships (Fig. 1A). Analyses of interspecific variation in microbiota in or on diverse body sites now frequently evaluate the presence or absence of phylosymbiosis. A growing number of examples of phylosymbiosis include the gut microbiota of *Nasonia* parasitoid wasps (1, 3), *Peromyscus* deer mice (2), American pikas (4), 23 different bat genera (5), dozens of mammalian species (6), and *Cephalotes* ants (7). Examples of phylosymbiosis also occur on the skin and surfaces of animals in aquatic environments including seven different *Hydra* species (8, 9), 20 sponge species (10), 21 major coral clades (11), and 44 tropical reef fish species (12).

It is important to highlight that phylosymbiosis is a measurable pattern that is assumption free with respect to process. First and foremost, it does not assume a stable evolutionary association of hosts and their microbiota or congruent splitting of ancestral, interacting organisms that may result from cospeciation or coevolution. However, it can be enhanced by such processes (11). Second, it does not assume that microbial communities are vertically transmitted. Rather, phylosymbiosis may be driven by vertical inheritance of the microbiota or persistent host filtering of acquired microbes, and it accommodates the possibility that host-microbiota associations could change each host generation due to various diets or environments (2, 13). Thus, phylosymbiosis is a pattern measured in a single instance of time and space, and it can theoretically change upon host exposures to a different community of environmentally acquired microbes.

As phylosymbiosis emerges as a bona fide, though not universal, trend in studies of diverse systems, a salient set of questions is emerging. What evolutionary forces (selection versus neutrality) underpin the pattern? What are the number and types of host and microbial genes that contribute to phylosymbiosis? Is phylosymbiosis more readily detectable in certain body sites or groups of host organisms? How consequential is phylosymbiosis to host biology? A model system with phylosymbiotic communities, host genetically tractability, interspecific interbreeding, axenic host rearing, and bacterial cultivability will be helpful in interrogating these long-term questions.

To test whether phylosymbiosis is consequential or not to host biology, microbiota transplantation experiments to germfree hosts can reveal whether an interspecific microbiota is helpful, harmful, or harmless relative to an intraspecific microbiota (Fig. 1B). For example, mice humanized with microbiota by oral gavage exhibit immunodeficiencies and increased susceptibility to enteric pathogens (14). However, transplants between distantly related hosts such as mice and humans cannot directly unravel the evolutionary processes and consequences that underpin phylosymbiosis. Comparisons between recently diverged species with phylosymbiotic microbiota are crucial for dissecting the consequences to host biology and selective pressures shaping phylosymbiosis.

We previously demonstrated that germfree *Nasonia* larvae exposed to interspecific microbiota suffered significant reductions in adult survival in comparison to intraspecific microbiota (2). However, that study did not reveal the developmental or survival defects in early life that precede and directly impact the reductions in adult survival. Moreover, crosses between two species of *Nasonia* lead to F2 hybrid death in haploid male larvae in conventionally reared hybrids and germfree hybrids exposed to microbiota but not in germfree hybrids (1). Thus, hybrid lethality is similarly contingent on the presence of gut bacteria, which suggests that hybrid breakdown results from costly host-microbiota interactions that are absent in the parental *Nasonia* species exhibiting phylosymbiosis.

The parasitoid wasp genus *Nasonia* (also known as the “jewel wasp”) is an evolutionary genetics model (15, 16) well suited for understanding the impacts of host-gut microbiota symbioses and recent speciation events because it is comprised of four interfertile species, N. vitripennis, N. longicornis, N. giraulti, and N. oneida (17, 18), that exhibit various degrees of reproductive isolation (19). The species *N. vitripennis* is estimated to have diverged from the ancestor of the three younger species 1 million years ago (mya). *N. longicornis* and *N. giraulti* diverged 0.4 mya, and *N. giraulti* and *N. oneida* diverged 0.3 mya (20, 21). Adult *Nasonia* female wasps lay their eggs within the puparia of Sarcophaga bullata flesh flies (17). Under laboratory conditions (constant temperature of 25°C), *Nasonia* has a short generation time of approximately 14 days, in which wasp metamorphosis and development occur within the *S. bullata* puparium. *Nasonia* affords an ease of conventional and germfree rearing (1, 22, 23), the ability to establish interspecies hybrids after curing of *Wolbachia* (24, 25), and the genetic advantages of haplodiploid sex determination, wherein males and females develop from unfertilized (haploid) and fertilized (diploid) eggs, respectively.

The *Nasonia* male microbiota is dominated by *Providencia* spp. and *Proteus* spp. during second instar larval development, typically representing 81 to 96% of the bacterial community. These bacteria are rod-shaped members of the Enterobacteriaceae family closely related to other intestinally associated insect bacteria such as *Escherichia* and *Enterobacter*. The dominant gut bacterial genus in Nasonia vitripennis and *Nasonia giraulti* larvae is *Providencia*, while the dominant gut genus in *Nasonia longicornis* larvae is *Proteus* (1). Notably in Drosophila melanogaster, several different *Providencia* strains induce variable levels of host immunogenicity (26, 27). This variability in immune response may provide a potential pathway by which these bacteria alter *Nasonia* development and survival.

Considering that much of the metabolic activity, growth, and development of *Nasonia* occur before adult eclosion, the functional effects of an interspecific microbiota will likely arise during the *Nasonia* life cycle following exposure in the larvae. In this study, we test the impacts of exposure of an interspecific microbiota on developing males using three *Nasonia* spp. and developmental stages spanning initial embryo hatching to adulthood and reproduction. The metrics analyzed include larval growth, pupation rates, and adult fertility and longevity. The microbiota treatment used for *Nasonia* exposure was heat inactivated in order to prevent bacterial overgrowth in the *Nasonia in vitro* rearing system that is also used for axenic rearing. The bacterial heat inactivation prevents *Nasonia* wasp exposure to microbiota under unchecked *in vitro* growth conditions. Heat-inactivated bacteria are commonly used as a means to probe a host’s immune response to bacterial stimuli (28–30) and metabolic rate (31, 32). Thus, this design tests the impacts of early-life exposure to inactivated microbiota on host responses during important periods of growth and metamorphosis.

## RESULTS

### Impacts of interspecific microbiota on larval growth.

**FIG 2:**
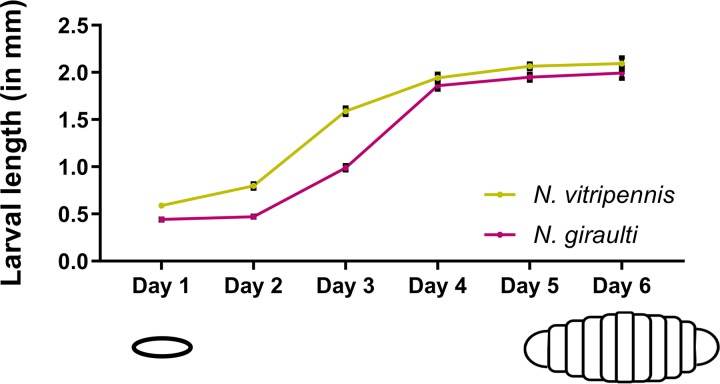
Peak larval growth occurs between the second and third instars 2 to 4 days after hatching. The average larval length of 1× PBS**-**treated *Nasonia* (medium control) is shown for the first 6 days of larval development. Days 1 and 2 represent the first two larval instars, days 3 and 4 represent the third larval instar after rapid growth, and days 5 and 6 represent the fourth larval instar before gut contents are evacuated for pupation. The yellow line displays *N. vitripennis* larval growth while the red line represents *N. giraulti* larval growth. Black bars at each developmental day indicate the larval length standard error of the mean (*n* = 13 to 15 per day).

To determine the effect of microbiota transplants on *Nasonia* larval growth, daily images of larvae exposed to interspecific microbiota from fourth instar larvae or phosphate-buffered saline (PBS) (negative medium control) were captured within their respective transwells until the larvae reached pupation. The lengths of all larvae in the transwells were measured using ImageJ software. Previous measurements of germfree Nasonia vitripennis larval growth demonstrated peak growth between day 2 and day 6 of larval development (22). Here, we benchmark additional growth metrics in the germfree state for both *N. vitripennis* and *N. giraulti* larvae. Figure 2 presents the growth curves of medium-control *N. vitripennis* and *N. giraulti* larvae. These curves confirm the period of largest larval growth during the second and third instars across slightly different time ranges for the two species. In *N. vitripennis*, peak larval growth occurs during day 2 to day 4 of larval development. There was a 1.14-mm increase in larval length between day 2 and day 3 of development, equating to 75% of the total larval growth before pupation. In *N. giraulti* larvae, the peak growth involving a 1.39-mm increase, which is 90% of the total larval growth, is slightly delayed and occurs between day 3 and day 4 of development. These results are generally consistent with the well-established observation in conventional rearing that *N. giraulti* takes an extra day in the life cycle to reach adulthood.

**FIG 3:**
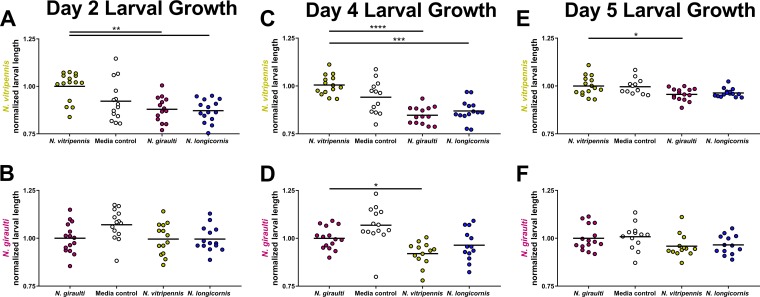
Interspecific microbiota slows larval growth. Normalized larval length for germfree *N. vitripennis* and *N. giraulti* exposed to heat-inactivated microbiota on day 2 (A and B) of larval growth before the second- to third**-**instar transition, day 4 (C and D) after most larval growth, and day 5 (E and F) of larval growth just before pupation. *x* axis labels represent the different microbiota transplant donor experimental groups. *, *P* < 0.05; **, *P* < 0.01; ***, *P* < 0.001; and ****, *P* < 0.0001, Kruskal-Wallis test with Dunn’s multiple correction.

With peak larval growth of *N. vitripennis* and *N. giraulti* established in the germfree rearing setup, the effects of interspecific, heat-inactivated microbiota were measured at the beginning and end of this growth. On day 2 of *N. vitripennis* larval growth after only 1 day of microbiota exposure, there was already a significant decrease in larval length upon exposure to an interspecific microbiota or medium control relative to the intraspecific microbiota (Fig. 3A). These results indicate that the costs of an interspecific microbiota are immediate and equal to or slightly stronger than the costs of being germfree. In contrast, on day 2 of *N. giraulti* larval growth, there was no significant difference in larval length between any of the treatment groups (Fig. 3B), perhaps because of the overall delayed larval growth in *N. giraulti*.

On day 4 of larval development at the end of peak growth, interspecific microbiota exposure causes a significant decrease in both *Nasonia* species. In *N. vitripennis*, the medium control trends lower but is not significantly different, whereas both interspecific microbiotas cause strongly significant reductions in larval length in the range of 12 to 15% average decreases. Interestingly, in *N. giraulti*, the only significant decrease in larval length occurred between the intraspecific transplant and the interspecific transplant from the most distantly diverged donor species, *N. vitripennis* (1 mya). These results suggest that the cost of exposure to an interspecific microbiota is impacted by the degree of phylosymbiotic differences in the microbiota. Only host recipients receiving a more distantly related microbiota suffer growth costs.

By day 4 of larval development, germfree *Nasonia* larvae have completed most of their growth (Fig. 2). To determine whether the growth costs of exposure to an interspecific microbiota persist beyond this period, measurements for day 5 were also recorded. As expected, the differences in larval length were now greatly diminished between the intraspecific and interspecific microbiota treatment groups for both host species. In *N. vitripennis* larvae, there was a single, significant difference with a small effect size. In *N. giraulti* larvae, there were no significant differences in normalized larval length on day 5 of development.

### Impacts of interspecific microbiota on pupation.

**FIG 4:**
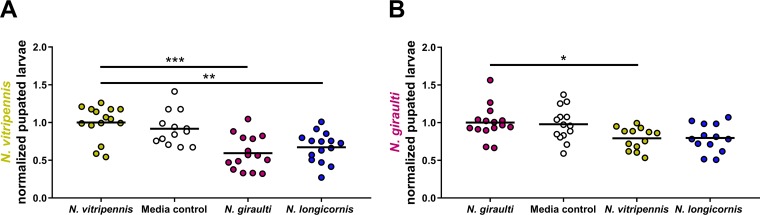
Early-life exposure to interspecific microbiota reduces pupation. Normalized proportion of pupated larvae on the last day of pupation before adult eclosion for germfree *N. vitripennis* (A) and *N. giraulti* (B) receiving medium or heat-inactivated microbiota. *, *P* < 0.05; **, *P* < 0.01; and ***, *P* < 0.001, Kruskal-Wallis test with Dunn’s multiple correction.

*Nasonia* pupation occurs over a 5-day period under germfree rearing and was assayed starting on day 10 of the total timeline in the experiment. It is important to note that during this stage, the developing wasps do not receive any additional rearing medium or microbiota exposure. In *N. vitripennis*, larval exposure to interspecific microbiota from *N. giraulti* and *N. longicornis* led to an average 40% and 32% decrease in pupation, respectively, in comparison to exposure to the intraspecific microbiota. Medium control had no effect on pupation (Fig. 4A). These decreases correspond to previously reported reductions of 43% and 23% from the same data set (2). In *N. giraulti*, there was once again a significant 21% decrease in pupation for the exposure to the interspecific microbiota from the more distantly diverged *N. vitripennis* donor (Fig. 4B), which in turn accounts for most of the previously reported 25% reduction in adult survival (2). There was also a 20% decrease in pupation of wasps exposed to the interspecific transplant group from *N. longicornis* (*P* = 0.06).

### Impacts of interspecific microbiota on adult fertility and longevity.

**FIG 5:**
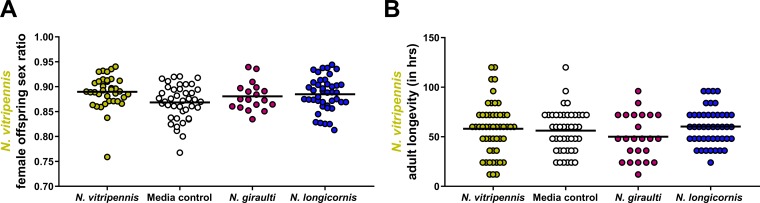
Early-life exposure to interspecific microbiota does not affect adult male fertility or longevity. (A) Adult male fertility is measured based on the female offspring sex ratio, which is normally maintained at ∼0.9 in conventional *N. vitripennis* rearing (40). Because of *Nasonia*’s haplodiploidy, a drop in this ratio represents a decrease in fertilization. (B) Adult male longevity is measured based on the number of living hours after mating with a female. Statistical analyses were performed using the Kruskal-Wallis test with Dunn’s multiple correction.

Adult males were moved from transwells to sterile mating chambers within 24 h after eclosion. The males from the different treatment groups were collected individually and mated to conventional *N. vitripennis* females. Male fertility was assessed by analyzing the female/male ratio for the resulting offspring because only fertilized embryos are females in *Nasonia* and other haplodiploid organisms. *Nasonia* sex ratios are normally female biased, and we observed normal sex ratios with no effect from early-life exposures to microbiota (Fig. 5A). Male longevity was recorded as the amount of time the males survived after mating without food or water. While there was a slight decrease in the longevity of the *N. vitripennis* wasps exposed to *N. giraulti* interspecific microbiota versus an intraspecific microbiota, there was no significant difference (*P* = 0.24) (Fig. 5B).

## DISCUSSION

Phylosymbiosis describes the ecoevolutionary pattern whereby the ecological relatedness (e.g., beta diversity relationships) of host-associated microbial communities parallels the phylogeny of the host species (1, 2). For the bacterial microbiota, phylosymbiosis is emerging as a widespread, though not universal, trend in a rising variety of animal holobionts (2, 4, 7, 11, 33, 34). This pattern was also recently extended, for the first time, to the virome in *Nasonia* parasitoid wasps (35). These results indicate that *Nasonia* evolution is associated with distinguishable microbiota and virus communities whose relationships recapitulate the wasp’s phylogeny. From this perspective, the microbiota or virome community composition is akin to a phylogenetic marker for the host species.

A key challenge in the study of phylosymbiosis is determining the evolutionary processes (selection or drift) that shape phylosymbiosis. For example, if the pattern results from inconsequential processes untethered to host fitness or performance, then hosts are not expected to benefit from a phylosymbiotic microbiota. However, if the pattern results from a selective pressure on hosts, then decreases in host fitness are expected upon exposure to interspecific or other nonnative microbiota. Selective pressures could drive the evolution of host traits that filter environmental microbiota in a specific way or facilitate vertical transmission of a host-associated microbiota. Selection on members of the microbiota to assemble in a phylosymbiotic manner may also enable the evolution of traits that optimize microbial fitness (e.g., replication) within the host. Higher microbial replication within the host may lead to increased rates of dissemination into the environment whereby members of the microbiota gain a (re)colonization advantage in the next host generation since there may be an increased likelihood of contact between hosts and microbes. This cycle between host-associated replication and environmental dissemination to other hosts could be a positive-feedback loop for microbial adaptations that contribute to the assembly of phylosymbiosis.

Here we report that early-life exposure of germfree *Nasonia* wasps to an interspecific heat-inactivated microbiota yields detrimental developmental and fitness impacts that begin early in larval development. After 3 days of daily exposure to heat-inactivated interspecific microbiota, larval growth delays in *N. vitripennis* and *N. giraulti* were most prominent during the point of peak growth in control wasps. Interestingly, in late larval development, the consequences of exposure to an interspecific microbiota subsided as larval size tended to equilibrate across the different treatment groups. However, comparable larval size this late in larval development may not be indicative of the energy storage needed at this developmental stage to successfully undergo pupation.

After microbiota exposure was halted during late larval development, recipient *N. vitripennis* larvae were significantly less likely to undergo pupation if they received interspecific microbiota from either of the more distantly diverged sister species. In *N. giraulti* recipient larvae, significant reductions in pupation occurred only with exposure to microbiota from their more distantly diverged sister species, *N. vitripennis* (1 mya), but not from the more closely related species, *N. longicornis* (400 thousand years ago [kya]). This disparity in *N. giraulti* development between the microbiota donors could shed light on the evolutionary timescale in which *Nasonia* wasp species develop a uniquely qualified and relatively beneficial microbiota. However, it is important to note that *N. giraulti* larval exposure to the *N. longicornis* microbiota did result in a reduction of pupation. Overall, these decreases in pupation from the interspecific microbiota directly translate to decreased adult survivability previously reported from the same experiment (2). By connecting *Nasonia* developmental delays with the resulting adult survival, we conclude there is a consequential impact of early-life exposure to interspecific microbiota on *Nasonia* development and fitness. Thus, phylosymbiosis is adaptive because the functional interactions between host and microbiota impact metamorphosis and therefore survival.

The *Nasonia* wasps were exposed with a normalized concentration of heat-inactivated microbiota from fourth instar larvae, so it is unlikely that the negative effects from an interspecific microbiota resulted from reduced nutrition conferred by the heat-inactivated microbiota, since they had comparable bacterial levels composed mostly of a few genera of *Gammaproteobacteria*. These microbiota exposures were also provided alongside a nutrient-rich *Nasonia* rearing medium (NRM) that, alone, has been sufficient to achieve *in vivo* levels of wasp growth and development. The *in vitro* rearing medium is made using the proteinaceous homogenate collected from *Sarcophaga bullata* pupae, the fly host of *Nasonia* wasps (23). In the process of making the *Nasonia* rearing medium, much of the pupae’s lipid contents are removed because they cause clogging in the sterile filtration process. Thus, we speculate that the interspecific differences in response to the medium control treatment group occur because *N. vitripennis* larval growth is slightly hampered by this lipid-limited environment, which is compensated by the addition of heat-inactivated microbes. *N. vitripennis* is a generalist species that parasitizes many different fly pupae in nature. Conversely, *N. giraulti* is a specialist on *Protocalliphora* bird fly pupae that are not reared in the lab. Thus, with our rearing medium, it is possible that a higher protein content artificially benefits germfree *N. giraulti* and results in a slight increase in larval growth, and this benefit could be negated upon exposure to microbes and the ensuing physiological changes that occur. While the medium control group had early larval growth trends, these nonsignificant growth differences disappeared by late larval development and did not influence the rest of the wasp life cycle whereas developmental differences from interspecific microbiota exposures were present through pupation.

We hypothesize that *Nasonia* larvae maintain a greater immune tolerance to intraspecific microbiota exposure than interspecific exposures. During larval development, the largest effects of the interspecific microbiota exposures occurred at a time when most of the larval energy supply was utilized on growth and storage. Any shunting of that energy flow into an activated immune response would presumably stunt growth (36), which was seen in Fig. 2. Prior studies indicate that *Drosophila* exposure to *Providencia* spp. can result in delayed larval growth and survival (26, 37). Moreover, in *Drosophila*, larvae that are unable to achieve adequate stores of lipids and other nutrients will be less likely to complete an energy-costly pupation (38, 39). However, additional experimentation is necessary to compare immune gene expression levels of key antibacterial pathways such as Toll and Immune Deficiency to show that interspecific microbiota transplants upregulate an immune response during larval development.

In *Hydra*, it has previously been observed that phylosymbiosis is in part regulated by species-specific antimicrobial peptide expression. Loss-of-function experiments demonstrated that distinct antimicrobial peptide compositions were necessary to maintain phylosymbiosis across multiple *Hydra* spp. (9). Selective pressures may similarly shape phylosymbiosis in *Nasonia* wasps through host immune pathways that curate and/or tolerate particular members of microbial communities over others. The impacts of selection on *Nasonia* phylosymbiosis are also supported by hybrid death and phylosymbiosis breakdown previously observed in *N. vitripennis* and *N. giraulti* F2 hybrid larvae. The loss of immune competence in these *Nasonia* hybrid larvae, as evidenced by hypermelanization and an overexpression of immune genes, coincides with 78% larval death and a marked shift in microbiota composition from the parent’s phylosymbiotic microbiota (1). Conversely, F2 hybrid larvae from the two younger species, *N. giraulti* and *N. longicornis*, do not melanize, die, or show shifts from the parental microbiota.

Beyond immunity, other mechanisms can play a role in the consequences of wasp-microbiota interactions such as metabolism, developmental signaling, and mate discrimination. For example, while there was no significant impact on adult reproductive capacity from the heat-inactivated transplants, it is possible that additional rounds of microbiota exposure during development could impact adult reproduction or perhaps adult mating behavior. In summary, this research reveals that early-life exposure of interspecific microbiota impacts larval growth and pupation within closely related species of the *Nasonia* model system. Further, by comparing the functional impacts of the gut microbiota across species that diverged between 400,000 and 1,000,000 years ago, we have shown that phylosymbiosis is not just indicative of a recent host phylogenetic effect on microbiota composition but is also important to traits involved in early-life host growth and fitness. Because *Nasonia* is a tractable animal system with interfertile species, it is an ideal model for testing the genetic basis of interconnections between development, microbial symbiosis, and speciation.

## MATERIALS AND METHODS

### *Nasonia* strains and collections.

*Wolbachia*-uninfected *N. vitripennis* AsymCx, *N. giraulti* RV2x[u], and *N. longicornis* NLMN8510 mated females were hosted on *S. bullata* pupae and housed in glass culture tubes capped with cotton at 25°C ± 2°C in constant light, as previously described (20). After 10 to 12 days, *S. bullata* puparia were opened, and virgin *N. vitripennis* or *N. giraulti* female offspring were collected as black pupae. Upon adult eclosion, 200 individual virgin females were isolated and provided two *S. bullata* pupae for 2 days of hosting to increase the egg deposition. As haplodiploids, *Nasonia* virgin females are fecund and lay all male (haploid) offspring. After the initial 2 days of hosting, females were provided with a new *S. bullata* pupa housed in a Styrofoam plug, allowing them to oviposit only on the anterior end of the host for easy embryo collection.

### Germfree rearing of *Nasonia*.

*N. vitripennis* AsymCx or *N. giraulti* RV2x[u] embryos were extracted with a sterile probe from *S. bullata* hosts parasitized by virgin females after 12 to 24 h. Twenty to 25 embryos per host were placed on a 3-μm-pore transwell polyester membrane (*n* = 12 to 15 transwells per treatment group) (Costar; Corning Incorporated, Corning, NY, USA) and sterilized twice with 70 μl of 10% bleach solution and once with 70 μl of 70% ethanol solution. The embryos were then rinsed three times with 80 μl of sterile Millipore water. After rinsing, the transwell insert was moved into a 24-well plate with 200 μl of NRM (prepared according to the NRMv2 protocol described in reference 23) in the basolateral compartment. All plates were stored in an autoclaved Tupperware box at 25°C ± 2°C under constant-light conditions for the duration of the experiment. Under sterile laminar flow, transwells were moved to new wells with 200 μl of fresh NRM every day. After 8 days, the transwells were drained of their medium on a sterile Kimwipe and moved to a clean, dry 24-well plate, and the 12 empty surrounding wells were filled with 1 ml of sterile Millipore water and 65.7 mM Tegosept solution to increase humidity and prevent fungal growth.

### Heat-inactivated microbiota preparation.

We tested the effects of interspecific microbial communities on host survival by transplanting heat-inactivated microbiota from three donor *Nasonia* species (*N. vitripennis*, *N. giraulti*, and *N. longicornis*) into *N. vitripennis* or *N. giraulti* male recipients. Microbiota were purified from fourth-instar larvae of each of the *Nasonia* donor species by homogenization of ∼100 larvae in 200 μl of sterile 1× PBS. The larval homogenate was then centrifuged at 800 rpm for 3 min to pellet large cellular debris, and the resulting supernatant was filtered through a 5- μm filter. The filtrate was centrifuged at 10,000 rpm for 3 min, and the supernatant was removed. The pellet was resuspended in sterile 1× PBS. This centrifugation step was repeated, and the pellet was resuspended in 200 μl 1× PBS. After the suspension was plated on tryptic soy agar to determine the rough microbiota concentration, it was heat inactivated by placement in a 75°C water bath for 1 h. After counting colonies on the tryptic soy agar plates, the heat-inactivated suspension was diluted in sterile 1× PBS to achieve a concentration of 5 × 10^6^ CFU of microbiota bacteria per milliliter. This procedure was performed independently for each of the donor microbial communities 1 day before exposure. Unfortunately, the current germfree rearing technique for *Nasonia* makes stable, live microbiota transplantation difficult to maintain. Because antibiotics and exogenous fetal bovine serum (FBS) were removed from the methodology to maintain a more natural system, the *Proteus* spp. and *Providencia* spp. have ample nutrients for and little resistance in the medium to overproliferating within the first 8 h after exposure. The issue lies with the stagnant environment of the *Nasonia* rearing medium within the transwell during *Nasonia* development.

### Transplantation of the heat-inactivated *Nasonia* microbiota.

After the first 24 h of germfree *Nasonia* rearing, the transwell inserts containing L1-stage larvae were randomly separated into four experimental groups for each recipient *Nasonia* genotype: a PBS negative control and three heat-inactivated *N. vitripennis*, *N. giraulti*, and *N. longicornis* microbiota exposure groups. For the transwell replicates in each group, 20 μl of this microbiota suspension was added directly to the transwell inserts daily during the eight transplantation days before *Nasonia* pupation. *Nasonia* rearing medium was replaced daily just before the inoculations. On days 2 to 8 of exposure, the transwell insert was drained on a sterile Kimwipe before the addition of the 20 μl of microbiota suspension. If there was any bacterial or fungal contamination in a transwell during the course of the *Nasonia* development, the transwell and its data were removed from the experiment.

### Comparative analysis of *Nasonia* development.

After the replacement of *Nasonia* rearing medium and addition of heat-inactivated *Nasonia* microbiota, a picture was taken of each well under magnification using a microscope-attached AmScope MT1000 camera. Pictures of the developing *Nasonia* in each transwell were taken from the first day of heat-inactivated microbiota exposure to the first day of adult eclosion (∼14 to 15 days). Starting on the second day of microbiota exposure, larval length was determined using ImageJ software by measuring the anterior-to-posterior end of larvae for all larvae in the transwell. Normalized larval growth per transwell sample was calculated as the average larval length of *Nasonia* per transwell divided by the average larval length of the intraspecific microbiota treatment group for each treatment day. Larvae were identified as dead if they were visibly desiccated or malformed and were not included in the analysis.

On the last day of *Nasonia* pupation before adult eclosion (5 days after initial pupation begins), the proportion of pupated larvae was measured for each transwell. Normalized pupation per transwell was calculated as the percent pupated *Nasonia* per transwell divided by the average percent pupated of the intraspecific microbiota treatment group.

For each transwell, live larval counts were recorded on the third day after embryo deposition to ensure the embryos hatched. Adult counts were determined by first recording the number of remaining larvae and pupae in each transwell 20 days after embryo hatching (5 to 7 days after first adult eclosion) and then subtracting that number from the larval counts previously recorded. Normalized adult survival per transwell sample was calculated as the percent survival of *Nasonia* from 3 days to 20 days after embryo hatching divided by the average percent survival of the intraspecific microbiota treatment group. Larvae and pupae were scored as dead if they were visibly desiccated or malformed. Larval growth, pupation, and adult survival between the intraspecific and interspecific treatment groups were compared using a Kruskal-Wallis test with a Dunn multiple-comparison test.

### Comparative analysis of adult reproductive capacity.

After initial adult eclosion from each experimental group, adult males were collected from the transwells daily and mated individually with conventional newly eclosed (<24-h) virgin females for 24 h. We measured adult male longevity by moving the individual males to a sterile glass after mating and recording the male survival status every 12 h. Adult males with deformities (walking impediments or deformed wings) that may influence mating were not used in the analysis. The mated female was then given two *S. bullata* pupae to parasitize. When the offspring pupae reached the black pupal development stage, the males and females were counted and a sex ratio was recorded.
